# Nutrient availability contributes to structural and functional diversity of microbiome in Xinjiang oilfield

**DOI:** 10.3389/fmicb.2024.1450226

**Published:** 2024-07-31

**Authors:** Wei Cheng, Wenzhuo Tian, Weilong Wang, Tianhua Lv, Tianqi Su, Mengmeng Wu, Yuan Yun, Ting Ma, Guoqiang Li

**Affiliations:** ^1^Key Laboratory of Molecular Microbiology and Technology, Ministry of Education, College of Life Sciences, Nankai University, Tianjin, China; ^2^Tianjin Engineering Technology Center of Green Manufacturing Biobased Materials, Tianjin, China

**Keywords:** IMEOR, community stability, community structure, nutrition, nitrogen cycle

## Abstract

Indigenous microbial enhanced oil recovery (IMEOR) is a promising alternative way to promote oil recovery. It activates oil recovery microorganisms in the reservoir by adding nutrients to the injected water, utilizing microbial growth and metabolism to enhance recovery. However, few studies have focused on the impact of injected nutrients on reservoir microbial community composition and potential functions. This limits the further strategic development of IMEOR. In this study, we investigated the effects of nutrition on the composition of the reservoir bacterial community and functions in the Qizhong block of Xinjiang Oilfield, China, by constructing a long core microbial flooding simulation device. The results showed that the microbial community structure of the reservoir changed from aerobic state to anaerobic state after nutrient injection. Reducing the nutrient concentration increased the diversity and network stability of the reservoir bacterial community. At the same time, the nitrogen metabolism function also showed the same change response. Overall, these results indicated that nutrition significantly affected the community structure and function of reservoir microorganisms. Injecting low concentrations of nutrients may be more beneficial to improve oil recovery. This study is of great significance for guiding IMEOR technology and saving costs at the field site.

## 1 Introduction

Indigenous microbial enhanced oil recovery (IMEOR) is a promising and sustainable method for oil recovery. The oil reservoir is a specialized ecological environment of high temperature, high salt, and high pressure that can be analogized to a giant bioreactor. Previous studies have described them as complex ecosystems filled with a variety of microbial entities (Orphan et al., 2001). The sustainability of the oil recovery functions and services provided by such ecosystems depends on a relatively stable microbial system, which is defined as the degree of change or turnover of the microbial community (Griffiths et al., 2008; Tripathi et al., 2018). IMEOR exploits the proliferation and metabolic capacity of microorganisms to increase oil production. The mechanisms of oil recovery mainly include biological blockage of large pores, changes of physical and chemical properties of crude oil, and an increase in the internal pressure of the reservoir. This approach incorporates biotechnology solutions to address the challenges of the oil industry (Sharma et al., 2023).

In the process of microbial oil recovery, nutrients are generally injected into the injection well in the oil field. However, there are often great differences in the microbial community structure and oil augmentation efficiency of different production wells in actual production. 16S rRNA gene analysis was used in a previous study to study the microbial community in a specific block of the North China Oil Field. Different microbial distributions are observed across producing wells in the same block, which correlated with different oil yields (Tang et al., 2012). Crude oil production is closely related to the structure of the reservoir microbial community (Yin et al., 2023). Nutrient injection is the primary means of IMEOR, which stimulates an increase in the abundance of dominant functional bacteria and inhibits harmful microbial species during oil extraction (Gao et al., 2018; Liang et al., 2022). For example, sulfate reducing bacteria (SRB), produce hydrogen sulfide gas which can corrode flood pipes in oil reservoirs and pose health hazards to workers. When the supply of nutrients is stopped, the relative abundance of hydrocarbon degrading bacteria decreased and the relative abundance of anaerobes increased (Wang et al., 2020). In the study of the effect of IMEOR on the microbial community and oil recovery rate in Luliang Oilfield, nutrient injection showed positive effects on aromatic hydrocarbon degradation, resin and asphaltene fractions, and the oil recovery rate increased from 51.2 to 60.7%. The difference in microbial community structure in different wells will lead to different oil recovery. In the field experiment at Shengli Oilfield, the oil recovery is increased by 9.14% by stimulating local bacteria with nutrient injection (Bao et al., 2009). The addition of nutrients to oilfield produced water in the laboratory shows the enhancement of microbial growth, microbial community transfer, and microbial degradation capacity (Li et al., 2020). Studies of individual IMEOR production wells at the field site have shown significant effects of nutrient injection on the diversity, composition and relative abundance of reservoir microbes (Wang et al., 2015). It can be seen that there is a close relationship between nutrition, microbial community structure, and oil recovery function. However, how the structure and function of reservoir microbial communities respond to nutrient injection has not been fully studied. This has a potentially important role in enhancing oil recovery.

The response mechanism of microbial communities to nutrient changes in the environment has been reported in other environments, and nutrient injection has significant effects on microbial communities in soil, wetland and ocean (Han et al., 2012; Li et al., 2019; Bannon et al., 2022). Globally, the addition of nitrogen-containing nutrients significantly affects soil microbial diversity and community structure (Wang et al., 2023). Resource availability is the key to controlling soil microbial diversity and the main factor regulating microbial functional traits (Zhu et al., 2023). In seawater sediments, microorganisms associated with nitrogen, phosphorus, and sulfur cycles show different abundances at different sites, and the metabolism of microorganisms is the main factor affecting the distribution of microbial communities (Lu et al., 2019). When affected by changes in external conditions, the transformation of carbon, nitrogen, phosphorus and sulfur will inevitably disrupt microbial homeostasis and lead to changes in the composition, diversity and metabolic function of the microbiome (Marzocchi et al., 2020). For example, “nitrogen metabolism” and “phosphate metabolism” are special metabolic functions that are critical to community stability (Xun et al., 2021). In oil reservoir ecosystems, the addition of appropriate concentrations of organic phosphorus and nitrogen can promote the production of biosurfactants by oil reservoir microorganisms (Shi et al., 2021). But how specialized metabolic functions in microbial communities change during this dynamic process has not been fully investigated.

To elucidate the response of microbial community structure and function to injected nutrients in a reservoir environment. We constructed a long core microbial flooding simulation device to simulate the whole process of IMEOR in Qizhong block of Xinjiang oil field. We continuously monitored for 60 days and sampled at 7 locations in the core. Based on 16S rRNA sequencing and metagenomic analysis, we studied the response changes of reservoir microorganisms to nutrient injection. And we focused on the link between different injected nutrient concentrations, microbial community stability and function. This study has important implications for cost savings in the field and for improving oil recovery.

## 2 Materials and methods

### 2.1 Medium and fluids

All fluids used in this study, including crude oil (the oil phase of the fluid produced in the wells) and produced water (the water phase of the fluid produced in the wells), were obtained from the Qizhong block oil field in Karamay, Xinjiang, China. The extracted water used in the experiment was mixed with formation water from five production wells. The sample well numbers were 72,602, 72,604, 72,605, 72,649, and 72,659. The samples were completely filled into 15 L sterilized drums, which were previously swept with nitrogen to prevent oxygen from diffusing into the samples. The samples were immediately transported to our laboratory. All freshly produced fluids were either used immediately or refrigerated at 4°C for subsequent experiments. The viscosity of the crude oil during the experiments was 5.55 mpa.s (thin oil, 37°C).

### 2.2 Long core microbial flooding simulation device

The device was constructed according to the previous method, as shown in Supplementary Figure S1 (Cheng et al., 2023). The porous medium in the tube was quartz sand (30–50 μm) with no pores. The measured permeability was 5,839 millidarcy (md). The formation water was fully saturated, with a calculated pore volume (PV) of 6,670 cm^3^. The entire process of the oil drive experiment was designed to simulate the entire oil recovery process of the field, including the water saturation stage, oil saturation stage, water drive stage, and microbial drive stage (tertiary oil recovery). The sand-filled tube was first saturated by formation water under vacuum conditions (water-saturated stage). Next, oil was injected into the sand-filled tube until the end of uniform flow of crude oil. After that, formation water was injected for the secondary extraction stage (water drive stage). The injection was stopped when the water content at the end of the sand-filled tube exceeded 98%. This was followed by a simulated microbial oil drive stage. The nutrient solution was injected for microbial oil recovery. All tests were maintained at a constant flow rate of 1 mL/min at 37°C for 60 days.

### 2.3 Sample collection

The nutrient infusion was carried out throughout the study according to the medium recipe in Table 1. The medium in the intermediate vessel was changed twice a day. Samples were taken from seven sampling ports. Each time the sampling port switch was turned on, the stored liquid was drained. Samples were collected in 5 mL centrifuge tubes and centrifuged immediately (12,000 r, 8 min). The precipitate was used for genomic DNA extraction, while the supernatant was used for the determination of nutrient concentrations. DNA extraction was performed using the AxyPre^TM^ Genomic DNA Miniprep Kit (Axygen Biosciences, CA, United States), the procedure of which was detailed in Supplementary material.

**Table 1:** Nutrient formulation and concentration for 60 days.

Injection time	Nutrient concentrations (g/L)			
1–40 days	Molasses 3.50	Corn extract powder 1.50	NH_4_Cl 4.0	(NH_4_)_2_HPO_4_ 3.0
40–60 days	Molasses 1.75	Corn extract powder 1.50	NH_4_Cl 2.0	(NH_4_)_2_HPO_4_ 1.50

### 2.4 Determination of nutrients

The supernatant of the extract was subjected to determination of nutrient concentrations. Total sugars (TS) were determined by the phenol-sulfuric acid method. Total nitrogen (TN) was determined using the persulfate oxidation method (Lima et al., 1997). Total phosphorus (TP) by the digestion-molybdenum-antimony method. The required kits were provided by Tianjin Hass Water Analytical Instruments Co. Detailed steps for the determination of the three nutrient concentrations were given in Supplementary material.

### 2.5 High-throughput 16S rRNA gene sequencing

Extract genomic DNA as described earlier (Gao et al., 2018). High-throughput sequencing of 16S rRNA genes was done by Beijing NovoGene Co. Qiime2 (Quantitative Insights into Microbial Ecology 2) was utilized to analyze the raw reads. After quality control, denoising, and chimera removal, sequences with 97% identity were clustered as one OTU. The representative sequence of each feature was compared with the Silva 132 database to ensure its taxonomy and with the NCBI 16S rRNA database to determine their best hits at the species level (Quast et al., 2012). The community structure of each sample was counted at the genus level. The abundance data has been rarefied at depth of 41,051 and samples were analyzed for α-diversity using the qiime diversity function by Qiime2. The prediction of functional genes for nitrogen and phosphorus metabolism was performed by PICRUST (version 1.1.0). The data presented in the study are deposited in the NCBI Sequence Reads Archive repository, accession number PRNA1021155.

### 2.6 Network construction

In this study, the OTU-OTU co-occurrence networks was initially constructed for 16, 20, 24, and 39 days (high nutrient concentration) and 41, 42, 53, and 58 days (low nutrition concentration). To avoid sparse OTUs affecting correlation analyses, OTUs from all samples were involved in the microbe-microbe interaction assessment after filtering out OTUs with a relative abundance <0.01%. Statistically robust correlations based on Spearman correlation coefficients (ρ) > 0.6 and corresponding *p*-values <0.01 were included in the network analysis, where each node represented a genus and each edge represented a significant correlation between two nodes (Zhu et al., 2019). The FDR method used for *p*-value adjustment. Network visualization and topology properties using R v 3.6.3 (‘psych’ package) and Gephi platform v 0.9.2^1^ (Chen et al., 2020). Topological coefficients, including the number of nodes and edges, average degree, graph density and negative correlation were calculated for each network to estimate the complexity of the network. We evaluated network stability at different injected nutrients concentrations by node residual ratio and robustness (Liu et al., 2023). The higher the remaining proportion of nodes in the network, the more robust it was and the more stable the microbial co-occurrence network was. Moreover, we constructed two nutrients-OTU co-occurrence networks to evaluate the relationship between the three nutrients (TS, TN, and TP) and OTU at high injected nutrients concentration and low injected nutrients concentration, respectively. The nodes unrelated to the three nutrients were removed. The method for constructing the nutrients-OTU co-occurrence networks is consistent with the method for constructing OTU–OTU networks.

### 2.7 Analysis of metabolic functions

We performed metagenomic sequencing on samples from days 7, 20, 39, 42, 48, and 53 of sampling port 10. Sequencing was done by Beijing NovoGene Co. We quantified the functions encoded by the microbial community in each sample according to a stratified search of HUMAnN2 (v 3.7), functionally annotated via the EggNOG (v 6.0) database (Hernández-Plaza et al., 2022), and stratified their relative abundance according to the genes that perform these functions. By applying traditional within-sample (alpha) and between-sample (beta) measures of community diversity to more accurately explore the contribution of species to the function of interest, defined here as the ‘diversity of contributions’ to the function (Franzosa et al., 2018). All computed gene categories were drawn from the broader categories of metabolic function, genetic information processing, organismal systems, and cellular information processing. Within these types, metabolic functions are further divided into broad metabolic functions and specialized metabolic functions (Xun et al., 2021). Previous studies had shown that the function of “nitrogen metabolism” (nitrification or denitrification) was limited to specific microorganisms (Chen et al., 2016). Narrow distribution of methanogenic and methane-oxidizing bacteria involved in the function of “methane metabolism” due to their aerobic/anaerobic requirements or through coupling with nitrogen and sulfuric metabolism (Zhu et al., 2015). The gene cluster related to “terpene and polyketide metabolism” consisted mainly of *Actinomycetes* and *Bacillus* from the soil microbial community (Crits-Christoph et al., 2018). Specialized metabolic functions in reservoirs included those related to ‘sulfuric metabolism,’ ‘nitrogen metabolism,’ and ‘methane metabolism,’ which had been shown to be restricted to specialized taxa (Yao et al., 2023). In this study, we focused on “nitrogen metabolism” and “phosphorus metabolism,” which had been shown that the nitrogen and phosphorus cycles affect the stability of the soil microbiome (Xun et al., 2021).

## 3 Results

### 3.1 Changes in nutrient concentrations and microbial communities

**Figure 1:**
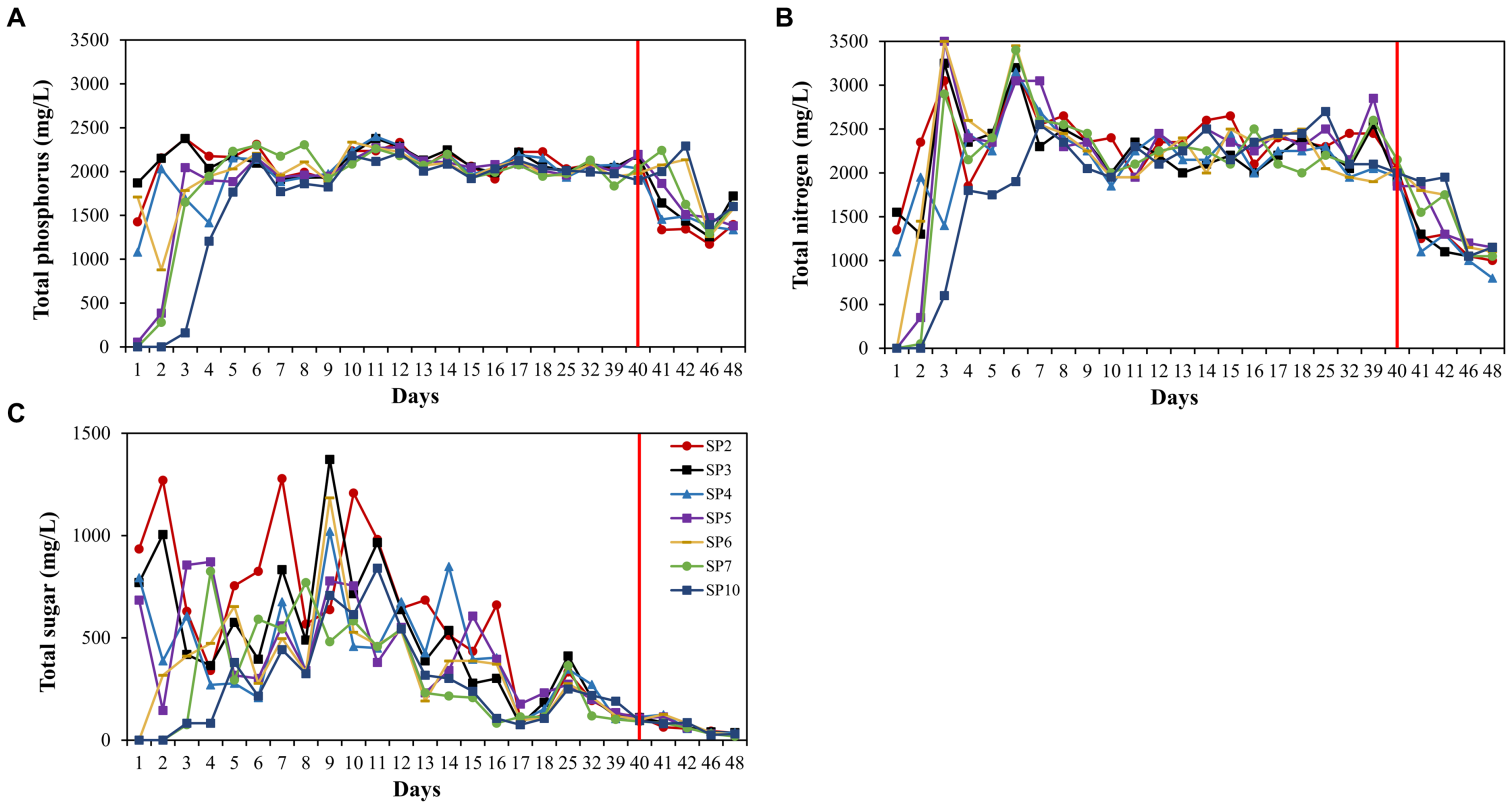
Nutrient concentration changes during the 60-day monitoring period. Concentration of total phosphorus **(A)**, concentration of total nitrogen **(B)**, and concentration of total sugar **(C)**. The sampling port 2, 3, 4, 5, 6, 7, and 10 are displayed with lines of different colors. SP2 represents sampling port 2, and so on for others.

Trends in total sugar, total nitrogen, and total phosphorus concentrations were consistent across the seven sampling ports, with a delay in nutrient concentration changes at the back end of the long core, but the delay was not perfectly consistent with the rate of injection (Figure 1). The change in the concentration of total sugars fluctuated more, and the concentration was maintained at 150 mg/L after 17 days (Figure 1C). Total nitrogen and total phosphorus stabilized after day 8 (Figures 1A,B). After 40 days, we lowered the concentration of injected nutrients and nutrient concentrations at each sampling port decreased.

We monitored changes in the microbial community at each sampling port for 60 days (Supplementary Figures S2–S5). When the injected nutrient volume was less than 1PV, the community structure of each sampling port was significantly different. We mainly focused on the changes in the dominant genus, *Exiguobacterium* was first activated after the nutrients reached each sampling port. *Exiguobacterium* belongs to the hydrocarbon oxidizing bacteria, which has the ability to degrade alkanes (Mohanty and Mukherji, 2008). The high growth of aerobic microorganisms may have contributed to the instability of the total sugar concentration in the pre-existing period. *Enterococcus* was a facultative anaerobic bacterium capable of producing organic acids through fermentation (Supplementary Figure S2), which was highly activated on days 6–10 (Schauf et al., 2019). After 12 days, some anaerobic fermenting genera were activated (Supplementary Figures S3, S4), such as *LNR_A2-18* and *Sphaerochaeta*, which were able to ferment to produce acetate, ethanol, hydrogen and CO_2_ (Ritalahti et al., 2012). The process of microbial community change was consistent with the results of our previous study (Cheng et al., 2023). This further demonstrated the succession of reservoir microorganisms from aerobic to anaerobic states during the indigenous microbial enhanced oil recovery.

### 3.2 Analysis of co-occurring network structure

A network analysis of the microbial community at each sampling port after nutrient injection was performed to determine if the microbial co-occurrence patterns changed over time. At the initial nutrient concentration, we performed network visualization of microbial communities on days 16, 20, 34, and 39. After lowering the injected nutrient concentration, we performed network visualization of microbes on days 41, 42, 48, and 53. The topological eigenvalues of microorganisms at both nutrient concentrations are shown in Table 2. Each network had unique topological properties. Overall, the injection of nutrients resulted in more nodes and edges in the microbial networks, higher average degrees, and more complex networks. This suggested that injection of nutrients increases the complexity of reservoir microbial networks. In each co-occurring network, positive interactions were greater than negative interactions.

**Table 2:** Topological eigenvalues of microbial network structure after nutrient injection.

Days	Node	Edge	Average degree	Density	Negative correlation
16	314	811	5.166	0.017	29.47%
20	323	1,114	6.898	0.021	35.73%
24	333	1,308	7.856	0.024	36.47%
39	379	1,275	6.728	0.018	27.06%
41	377	1,227	8.509	0.017	33.58%
42	380	1996	10.505	0.028	12.88%
48	372	3,002	16.14	0.044	38.04%
53	409	1743	8.523	0.021	22.83%

**Figure 2:**
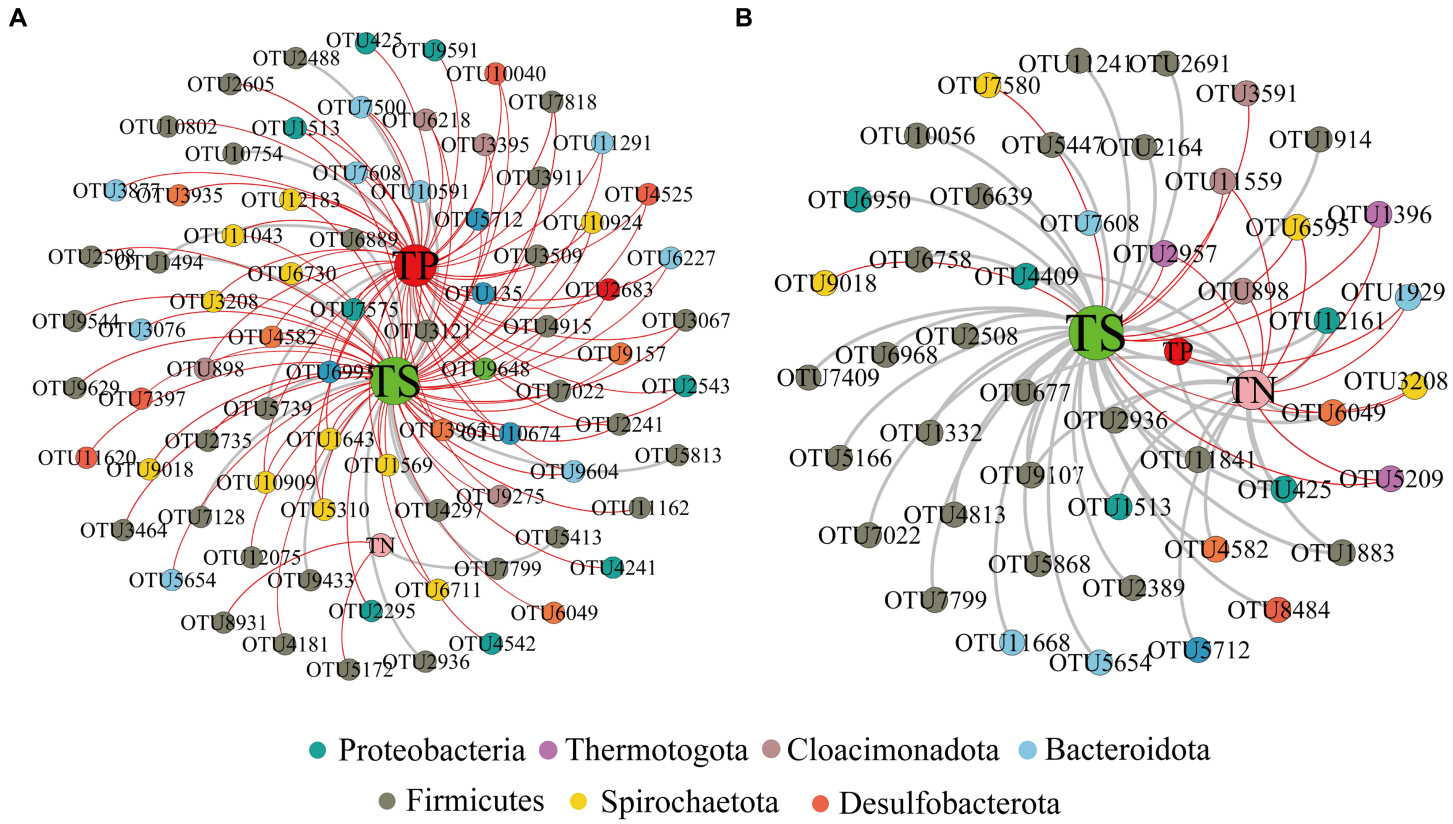
Network visualizes the nutrient-OTU interactions between initial nutrient concentration **(A)** and reduced nutrient concentration **(B)**. Positive correlations are shown in gray and negative correlations in red. The size of each node is proportional to the betweenness centrality. The red, green and pink dots marked with TP, TS and TN represent total phosphorus, total sulfur and total nitrogen, respectively.

In addition, we constructed a total network based on OTU-Nutrition (Figure 2). After removing unconnected nodes, the total network at the initial nutrient concentration consisted of 82 nodes and 124 edges (16% of positive edges). Microorganisms connected to TP and TS by microbial annotation of OTUs in the network included Proteobacteria, Firmicutes, Bacteroidota, Cloacimonadota, Spirochaetota, Desulfobacterota, and Thermotogota. Microorganisms connected to TN include *Firmicutes* (Figure 2A).

After reducing the nutrient concentration, the total microbial network consisted of 49 nodes and 68 edges (72% of positive edges) (Figure 2B). Microorganisms linked to TS include Proteobacteria, Firmicutes, Cloacimonadota, Spirochaetota, Desulfobacterota and Thermotogota. Microorganisms linked to TP include Proteobacteria and Bacteroidota. And microorganisms linked to TN include Proteobacteria, Firmicutes, Bacteroidota, Cloacimonadota, Spirochaetota, Desulfobacterota and Thermotogota. The relationship between nutrients and microorganisms together constitutes the ecological network of reservoir microorganisms.

### 3.3 Stability of co-occurring networks

**Figure 3:**
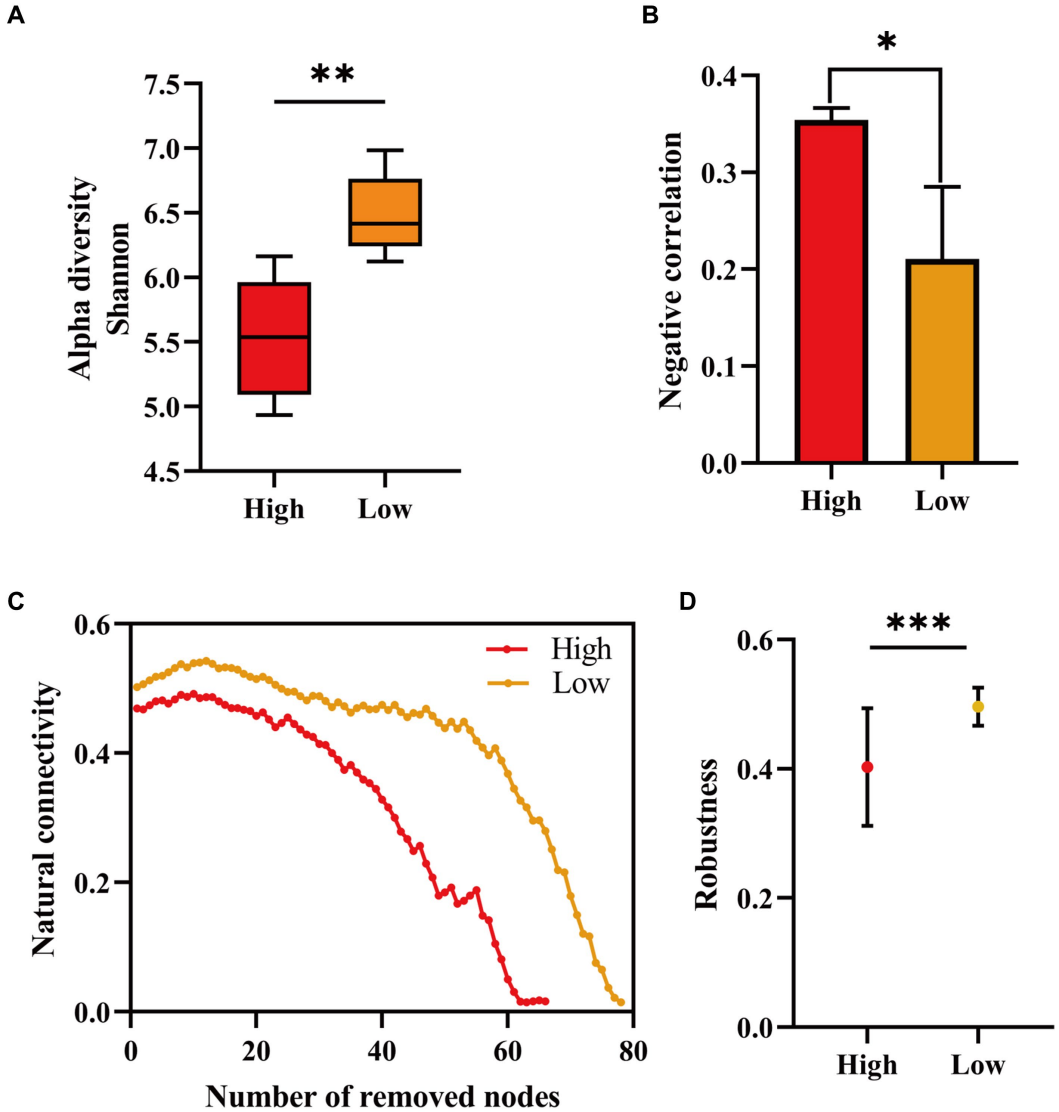
Microbial α-diversity at different injected nutrient concentrations **(A)**, proportion of negative correlation in the co-occurring network **(B)**, proportion of remaining nodes in the network after randomly deleting some nodes **(C)**, and proportion of remaining nodes in the network after randomly deleting 50% **(D)**. Initial injected nutrient concentration stage (high) and reduced injected nutrient concentration stage (low). Alpha diversity, negative correlation, and robustness are significant at different stages of injected nutrient concentration (*p < 0.05, **p < 0.01 and ***p < 0.001).

We counted the proportion of negative correlations in community co-occurrence networks under high (initial nutrient concentration) and “low nutrients,” and the microbial network structure showed lower negative interactions after lowering the nutrient (Figure 3B). The results showed that the negative interactions among reservoir microorganisms was weakened after lowering the nutrients. Also our results showed an increase in bacterial diversity after decreasing the injected nutrient concentration (Figure 3A). Low nutrient concentrations may increase the diversity of bacterial communities (Yun et al., 2021). We evaluated the stability of the microbial network before and after nutrient reduction. The proportion of remaining nodes in the network after randomly removing some nodes was calculated (Figure 3C). We also counted the percentage of nodes remaining in the network after randomly deleting 50% of the nodes (Figure 3D). The results all indicated that the network structure of reservoir microbial communities showed higher stability after reducing nutrients.

### 3.4 Changes in the potential functions of reservoir microbial communities

**Figure 4:**
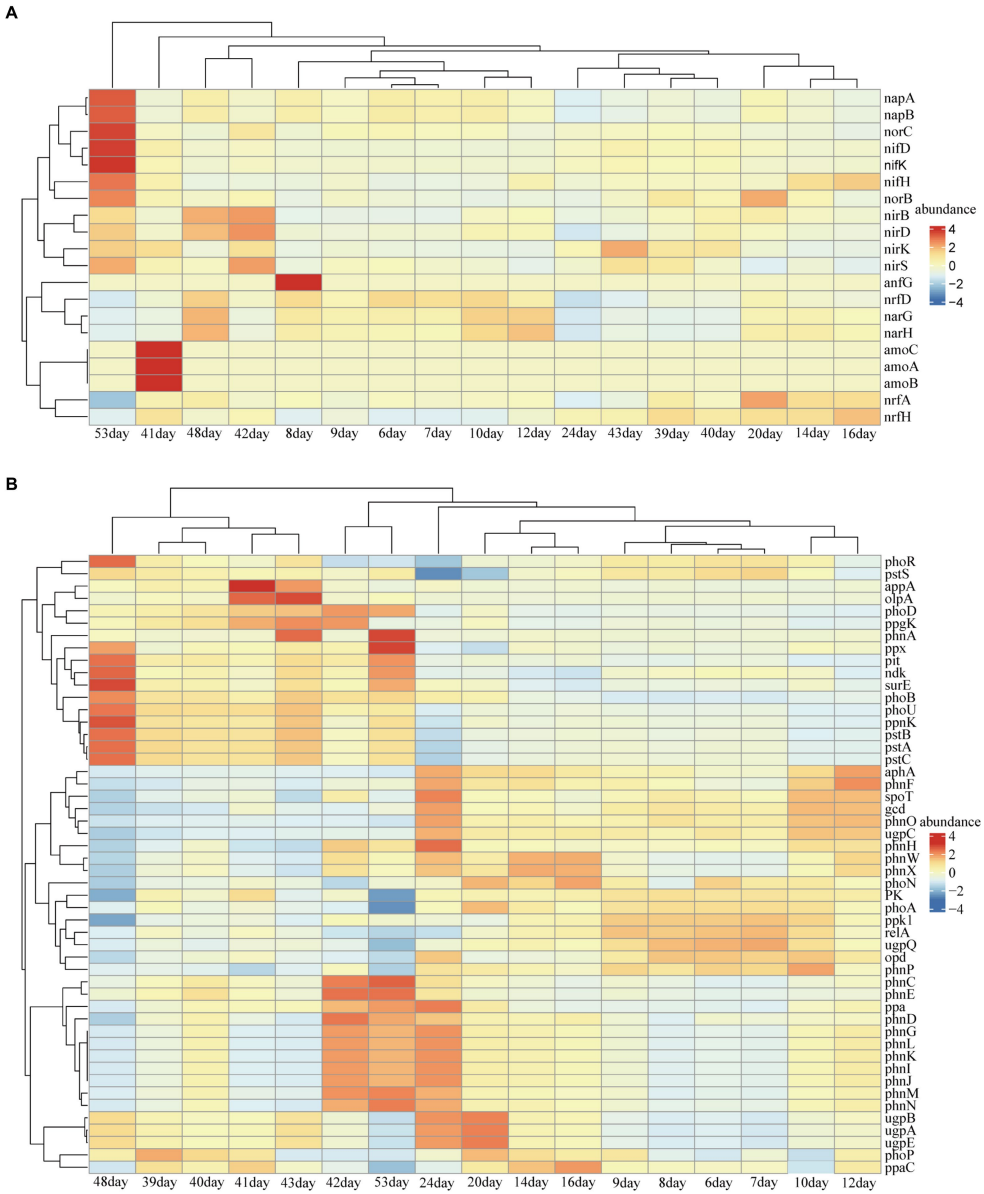
Predicted abundance of functional genes for nitrogen **(A)** and phosphorus **(B)** metabolism obtained from PICRUSt results. Less than 1% of functional genes and unclassified functional genes were removed.

**Figure 5:**
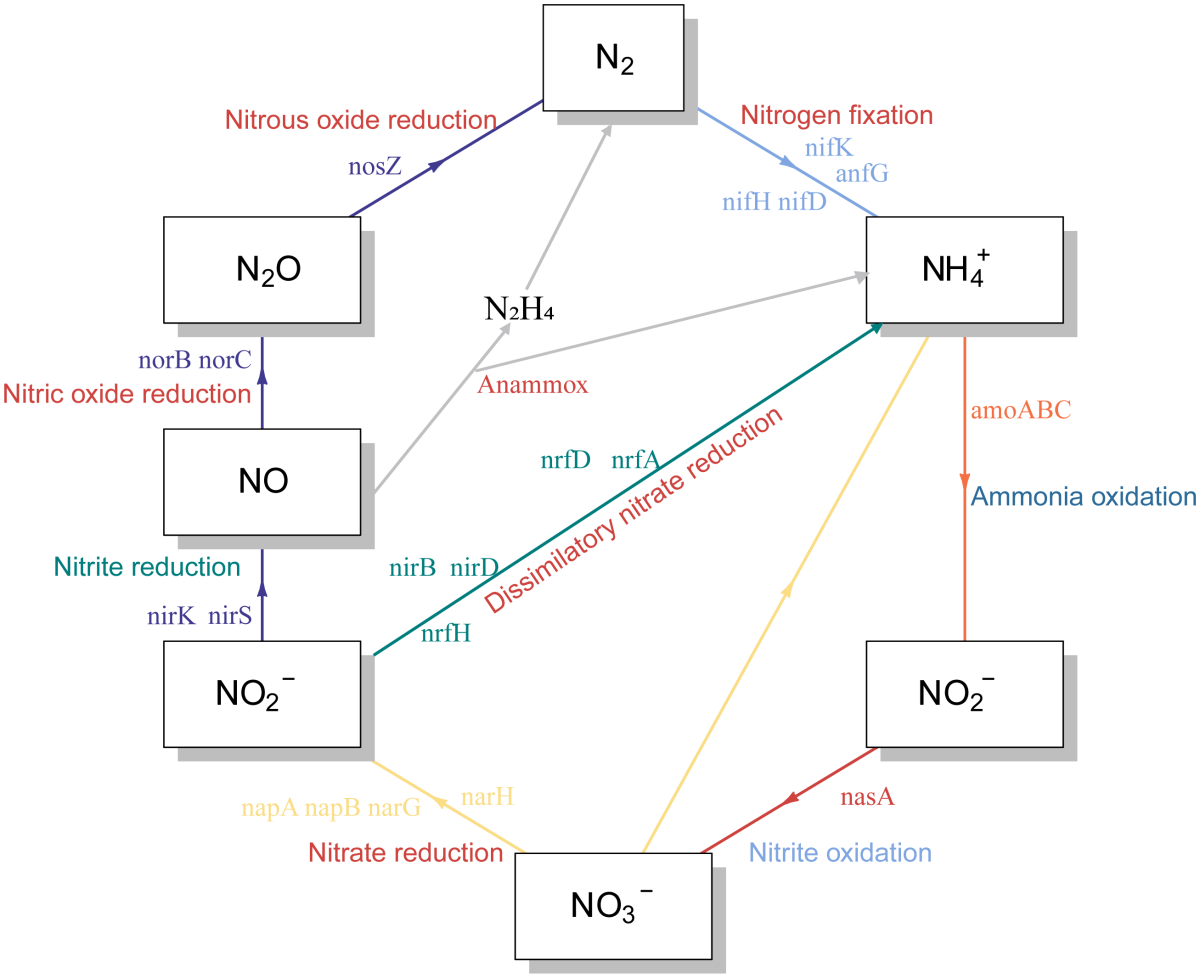
Predictions of nitrogen cycle metabolic pathways from PICRUSt results.

Reduced nutrition had a positive effect on the stability of the reservoir microbial network, but the effect on nitrogen and phosphorus-related metabolic functions in the community was unknown. Therefore, we predicted the abundance changes of related functional genes based on PICRUST. Notably, the abundance of functional genes related to nitrogen metabolism increased after lowering the injected nutrient concentration, especially on days 41, 42, 48, and 53 (Figure 4B). These metabolic processes included nitrogen fixation, nitrite reduction to ammonia, ammonia oxidation, nitrate reduction, dissimilatory nitrate reduction and nitric oxide reduction (Figure 5). Many microorganisms related to nitrogen metabolism have a potential role in improving crude oil recovery, such as NRB and denitrifying bacteria (Niu et al., 2020). Our results suggested that injecting low concentrations of nutrients may be more beneficial for improving oil recovery.

**Figure 6:**
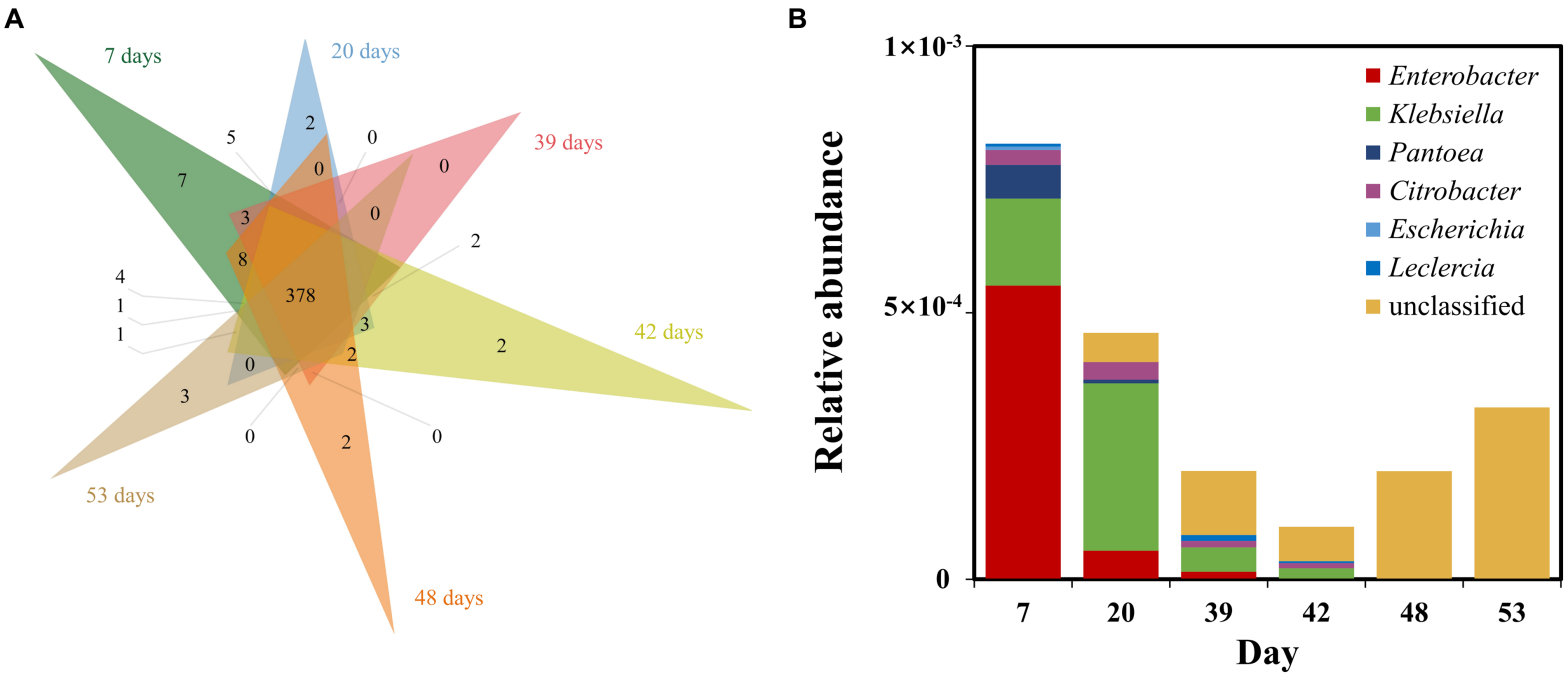
Venn diagram showing the types of microbial metabolism detected at different stages of injected nutrient concentration at sampling port 10 **(A)**. Diversity of contributions within samples of nitrogen metabolism and phosphorus metabolism pathways **(B)**.

**Figure 7:**
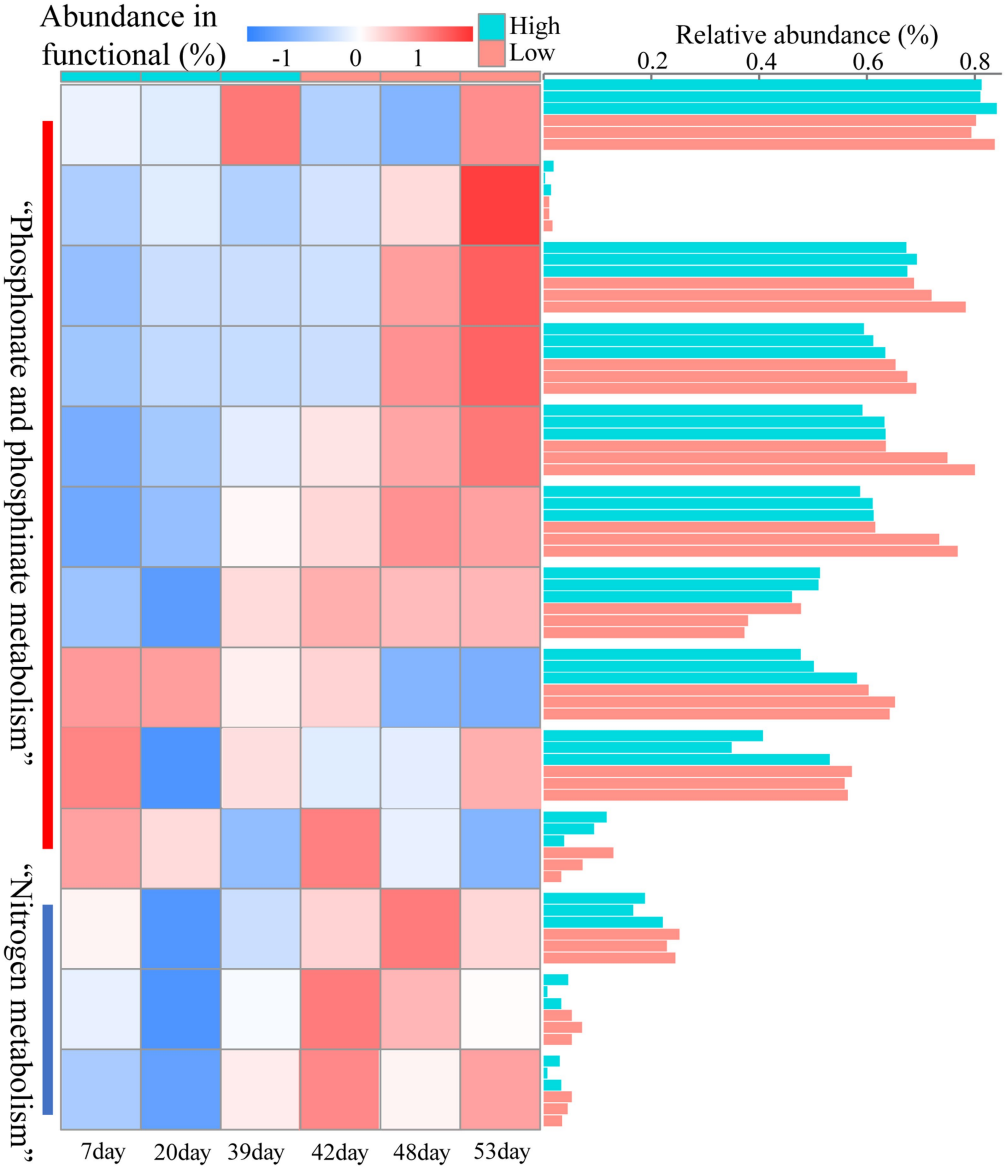
Heatmaps of the relative abundance of nitrogen metabolism and phosphorus metabolism at different injected nutrient concentrations obtained based on metagenomic results.

We performed metagenomic sequencing of low and high nutrient samples from sampling port 10, and further analyzed nitrogen metabolism and phosphorus metabolism functions in the samples by performing stratified searches using the HUMAnN2 tool (Franzosa et al., 2018). The total mapped abundance to EggNOG database is within the range of 47.30–61.73% (Supplementary Table S1). The major community composition identified by HuMAnN2 were *Methanocorpusculum*, *Vibrio*, *Methanosarcina*, etc. (Supplementary Table S2). The results showed that the types of metabolic functions were similar in the six samples (Figure 6A). The metabolic types involved in phosphorus metabolism were methylphosphonate degradation I, inosine-5′-phosphate biosynthesis and pentose phosphate pathway, etc. The types of metabolism related to the nitrogen cycle included nitrate reduction (assimilatory) and nitrate reduction (denitrification). Notably, the relative abundance of nitrogen metabolism in the microbiome was higher at high nutrient concentrations compared to low nutrient concentrations (Figure 7). This was consistent with predicted changes in the abundance of functional genes for nitrogen metabolism (Figure 4A).

In addition, we further explored the diversity of contributions in reservoir microbial nitrogen metabolism pathways (functions with broad “unclassified” abundances may be contributed by one or more different species within or between samples, and thus they were excluded from this analysis) (Figure 6B). The genera that made a major contribution to reservoir nitrogen metabolism mainly include *Enterobacter*, *Klebsiella*, *Pantoea*, *Citrobacter*, *Escherichia*, and *Leclercia*. Contributors to nitrogen metabolism were variable across samples, further demonstrating the potential inconsistency between individual functions and community-level diversity. Although nitrogen metabolism had complex and variable contributors in each sample, both *Enterobacter* and *Klebsiella*, which were major contributors, belonged to *Proteobacteria* (Carter et al., 2004; Manter et al., 2011).

## 4 Discussion

In order to explore the response of microbial community structure and function to nutrition. In this study, a long core microbial flooding simulation device was constructed under laboratory conditions and dynamically monitored for 60 days. The effects of nutrition on microbial community composition, network stability and function were explored. At the beginning of the injection of nutrition, the community microorganisms were different from each sampling port due to the delayed nutrition (Supplementary Figure S2). As nutrients gradually arrived at each sampling port, the difference of microbial community structure gradually decreased. This is also the case in oilfield sites, where the interwell connectivity of the reservoir media is complex, and the pore size of different reservoir rocks also has a filtering effect on the injected nutrients (Dong et al., 2023). Injected nutrients may be consumed by the time they reach each well, resulting in differences in microbial community structure between wells. This suggests that the level of nutrient supply may be the main factors affecting the microbial community structure of different production wells in the same block.

We observed changes in the dominant genus and could find that the hydrocarbon oxidizing bacterium (*Exiguobacterium*) would be activated first (Supplementary Figure S2). On days 6–20, *Enterococcus* became the main dominant genus (Supplementary Figure S3). After 20 days, some anaerobic fermenting genera gradually gained some dominance, such as *LNR_A2-18* and *Sphaerochaeta* (Supplementary Figure S4). They belonged to the anaerobic fermenting bacteria, which was consistent with the hypothesis of microbial ecological “food chain” succession in the reservoir, which also tentatively supported the two-step activation theory of the transition from aerobic to anaerobic microorganisms in the reservoir (Cheng et al., 2023).

Network analysis helps to gain insights into the interactions between microbiomes and reveals patterns of co-occurrence between microorganisms. After nutrient injection, topological features showed that the network became more complex, suggesting that nutrient injection led to stronger connections among microorganisms. The positive interaction among reservoir microorganisms was dominant. Secondly, the microbial community structure showed higher bacterial diversity and lower competition after lowering the injected nutrient concentration (Figures 3A,B). During oil recovery, only a few groups of bacteria with specialized functions are enriched, such as hydrocarbon oxidizing and nitrate-reducing bacteria, other bacteria with broad functions or smaller numbers are reduced or even disappear, which can lead to fewer species and lower α-diversity in the ecosystem (Yun et al., 2021). Increasing bacterial diversity improves the functional redundancy of the reservoir microbiome, which is conducive to increasing oil recovery potential. In the gut microbial system, loss of bacterial diversity had been observed to enable microbial community shifts and associated loss of metabolic functions (Shang et al., 2021).

Microbiome stability can be defined in terms of community resilience (the ability of the community to return to a relatively stable state) or resistance (the ability of the community to resist external disturbances) (Allison and Martiny, 2008). We provided valuable insights into the stability of microbial communities by assessing the efficiency of reservoir microbial networks and the loss of nodes after simulating nutrient injection. Although most previous studies had not directly examined stability, less connected network structures were thought to reduce microbial community stability (Mougi and Kondoh, 2012; Zhang et al., 2023). We assessed the stability of the network by randomly removing nodes, and the microbial network showed higher stability after reducing the nutrient concentration (Figures 3C,D). The level of nutrient availability influenced the stability of microbial communities, and “high nutrients” seemed to negatively affect the stability of reservoir microbiomes. These results suggested that in reservoir microbial systems, high nutrient concentrations may increase negative interactions between bacteria, leading to a loss of bacterial diversity and a decrease in microbial community stability, which may affect reservoir microbial oil recovery capacity (Wang et al., 2023). Therefore, in the oilfield field, the selection of the concentration of injected nutrients is particularly important. If a suitable low-nutrient formulation is selected for injection and extraction, it will improve the crude oil recovery and reduce the cost investment at the same time.

It has been suggested that unstable microbial network structures reduce the number of functions provided by the community (Wei et al., 2015). In soil ecosystems, nitrogen metabolism has been found to be closely related to the stability of microbial networks (Xun et al., 2021). By predicting the abundance of nitrogen cycle-related genes at the 16 s level, which were dominated by nitrate reduction (Figures 4A, 5). This phenomenon was beneficial for oil recovery because nitrate-reducing bacteria can control acidification and protect metal pipelines through biocompetitive rejection of SRBs in reservoirs (Duque et al., 2004). Increased abundance of genes functioning in nitrogen metabolism after reduced nutrition (Figure 4). An elevated abundance of associated nitrogen metabolism was also observed in the metagenomic results (Figure 7). Previous studies suggested that nitrogen metabolism may be a key metabolic function in maintaining microbial community stability (Xun et al., 2021). Our results also showed that nitrogen metabolism and related functional genes showed the same response to changes in microbiome network stability. The diversity of contributions to key functions of “nitrogen metabolism” suggested that some of the major bacterial genera (*Enterobacter* and *Klebsiella*) were the main microbes involved in nitrogen metabolism (Figure 6B). They may be key species associated with microbiome stability. *Enterobacter* and *Klebsiella* belong to the Proteobacteria, a group of nitrogen-fixing bacteria. In the results of the total network of OTU-nutrition, microorganisms connected to TN were also found to contain *Proteobacteria* (Figure 2). Previous studies based on network analysis of 16S rRNA gene amplicon sequencing data had shown that most of the key taxa of bacteria belonged to *Proteobacteria* (Ma et al., 2016). Not surprisingly, specialized metabolic functions of nitrogen metabolism in reservoirs may be related to reservoir microbial community stability, containing a key group of microorganisms belonging to *Proteobacteria*. Many genera of *Proteobacteria* have the ability to enhance crude oil recovery (Yao et al., 2023). Therefore, the nutrient concentration, microbial community stability, and functional oil recovery bacteria that are intrinsically linked to each other should be focused on in future research.

In this study, the effect of nutrients on the structure and function of reservoir microbial communities was investigated by a long core microbial flooding simulation device. This provides new insights into the concentration selection of nutrients injected into the field, and injecting low concentration nutrients may be more beneficial to improve oil recovery and save oil field costs. Since reservoir microorganisms in specific blocks were selected in this study, it does not represent the overall changes in microbial communities in all oilfields. In addition, we mainly explore the nitrogen metabolism function in reservoir microorganisms, and more extensive research is needed in the future for other functions (carbon metabolism, sulfur metabolism, and methanogenesis), especially for the understanding of reservoir microbial communities and functional adaptations. This will facilitate a better understanding of the effects of nutrition on reservoir microbial community structure and function during microbial oil recovery.

## 5 Conclusion

In this study, we investigated the effects of nutrient injection on the structure and function of reservoir microbial communities by constructing a long core microbial flooding simulation device. The study showed that the injection of nutrients made the microbial network structure more complex, the microbial community structure underwent obvious turnover, and the reservoir microbial community transitioned from aerobic to anaerobic states. Reducing the injected nutrient concentration increased the diversity, network stability, and functional abundance related to nitrogen metabolism in the reservoir bacterial community. Overall, nutrient availability significantly affects the composition and function of the reservoir microbiome. In the field, the injection of low concentrations of nutrients may be more beneficial to improve oil recovery. Future studies should continue to explore the relationship between nutrient concentration and oil recovery function and elucidate the changes in microbial community function after nutrient injection. This study provides new insights into reservoir microbiota and ecosystem functions as well as guidance for field applications in oil fields.

## Data availability statement

The data presented in the study are deposited in the NCBI repository, BioProject accession number PRJNA1131017.

## Author contributions

WC: Conceptualization, Data curation, Investigation, Methodology, Software, Writing – original draft. WT: Formal analysis, Methodology, Project administration, Supervision, Validation, Writing – review & editing. WW: Data curation, Methodology, Writing – review & editing. TL: Methodology, Project administration, Supervision, Writing – review & editing. TS: Formal analysis, Methodology, Supervision, Writing – review & editing. MW: Methodology, Supervision, Writing – review & editing. YY: Conceptualization, Formal analysis, Investigation, Project administration, Validation, Writing – review & editing. TM: Funding acquisition, Resources, Visualization, Writing – review & editing. GL: Funding acquisition, Resources, Visualization, Writing – review & editing.

## Footnotes

^1^https://gephi.org/
