# Supplementary material for: Nutrient availability contributes to structural and functional diversity of microbiome in Xinjiang oilfield
Source: Front Microbiol. 2024 Jul 31;15:1450226. doi: 10.3389/fmicb.2024.1450226 (PMC11322141; doi:10.3389/fmicb.2024.1450226)
Supplement: Supplementary file 1 [file Image_1.PDF]

Nutrient availability contributes to structural and functional diversity of microbiome in Xinjiang oilfield

Wei Cheng[a,1], Wenzhuo Tian[a,1], Weilong Wang[a], Tianhua Lv[a], Tianqi Su[a], Mengmeng Wu[a], Yuan Yun [a*], Ting Ma[a,b*], Guoqiang Li[a,b*]

[a]Key Laboratory of Molecular Microbiology and Technology, Ministry of Education, College of Life Sciences, Nankai University, Tianjin, China
[b]Tianjin Engineering Technology Center of Green Manufacturing Biobased Materials, Tianjin, China

***For correspondence author:**
E-mail: yuanyun@nankai.edu.cn.
E-mail: tingma@nankai.edu.cn, Tel. 86-22-23508870.
E-mail: gqli@nankai.edu.cn, Tel. 022-23498185.

Wei Cheng and Wenzhuo Tian contributed equally to this work. Author order was determined on the basis of seniority.

1. Supplementary Figures

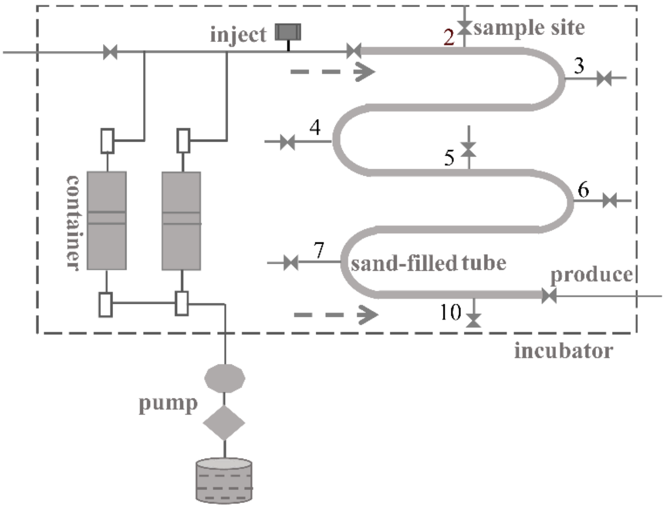

Fig. S1 A scheme of the long core microbial flooding simulation device.

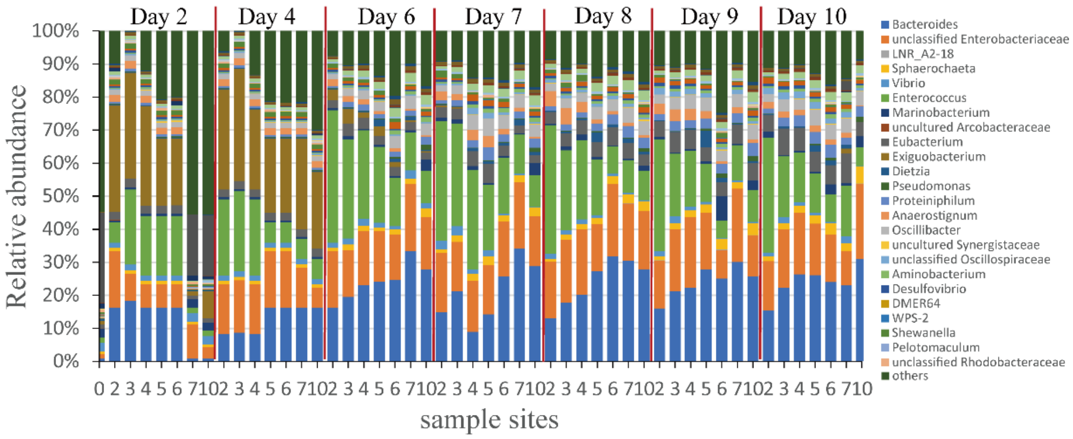

Fig. S2 Bacterial composition changes at the genus level in the seven sampling points (0-10 day).

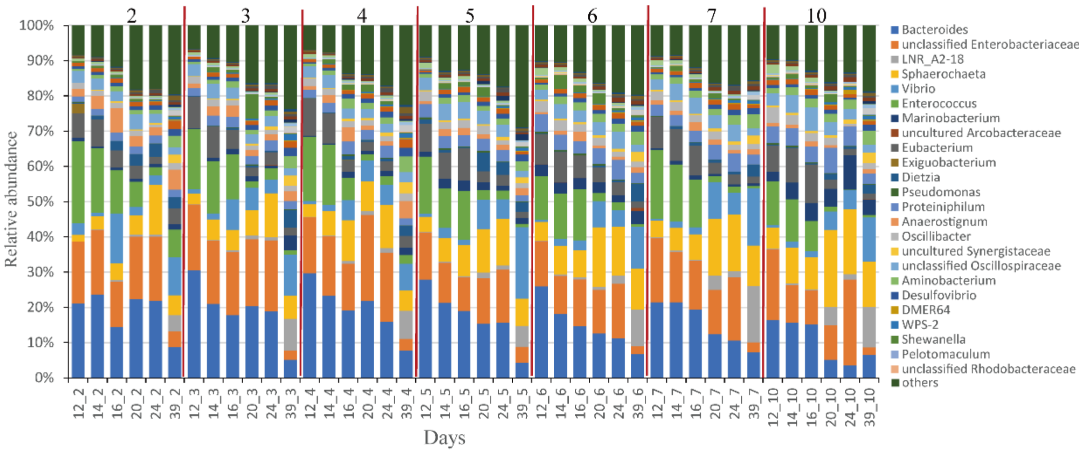

Fig. S3 Bacterial composition changes at the genus level in the seven sampling points (10-40 day).

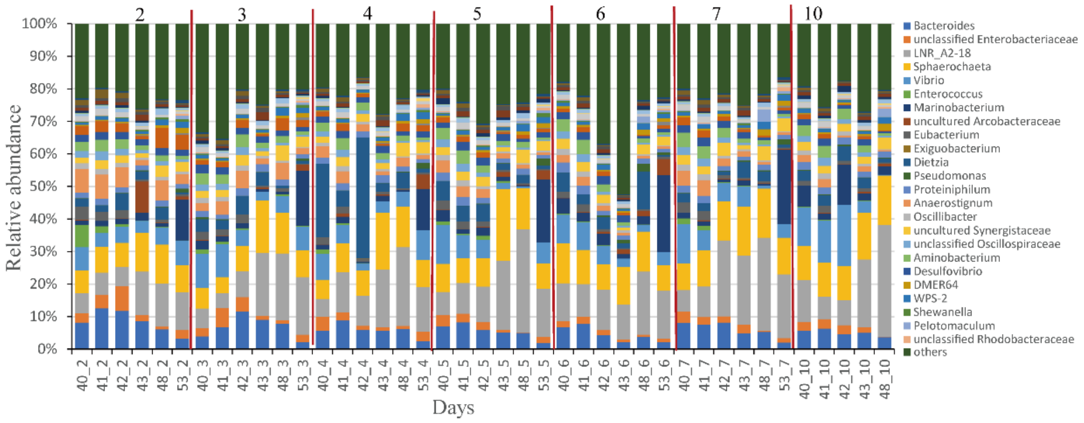

Fig. S4 Bacterial composition changes at the genus level in the seven sampling points (40-60 day).

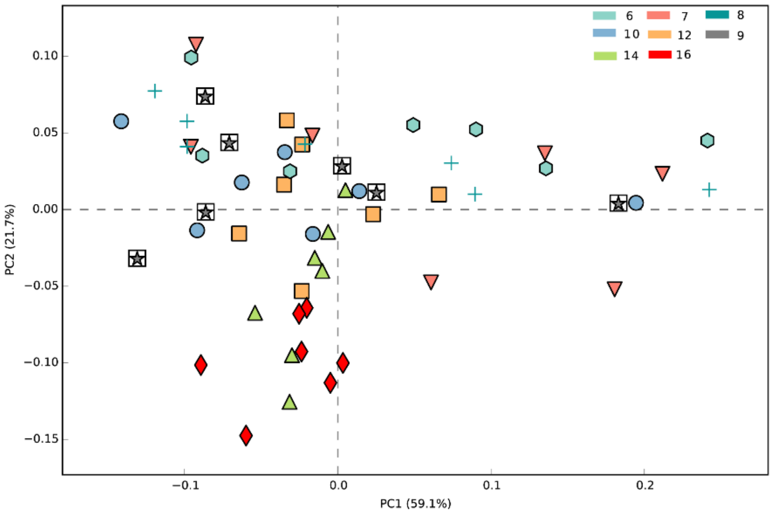

Fig. S5 Principal Component Analysis of microbial communities at different sampling points (first 10 days).

2. Supplementary methods

2.1 The total nitrogen content in the samples was determined by persulfate oxidation.

(1) Open the DBR digester and heat it to 105 °C.
(2) Add total nitrogen persulfate reagent into the low range digestion reagent tube.
(3) Sample determination: Add 2 mL sample into a reagent tube (estimate the sample concentration and dilute it with deionized water in the kit to the measurement range). A 2 mL kit of deionized water was used as a blank control.
(4) Cover and shake vigorously for 30 seconds.
(5) Insert the test tube into the digestion device and heat digestion for 30 minutes.
(6) Remove and cool to room temperature after digestion.
(7) Add total nitrogen A reagent.
(8) Cover and shake for 15 seconds and let stand for 3 minutes.
(9) At the end of the reaction, total nitrogen B reagent was added.
(10) Cover, shake the reagent tube up and down violently for 15 seconds, and let it stand for 2 minutes.
(11) After the reaction, open the total nitrogen C reagent tube and add 2 mL digestion solution.
(12) Cover and shake for 10 seconds. Let stand for 5 minutes.
(13) The time is over and the reading is measured.

2.2 Determination of total phosphorus in samples by digestion-Molybdenum-antimony method.

(1) Open the DBR digester and heat it to 150 °C.
(2) Add 5 mL sample into the total phosphorus reagent tube with a pipette (estimate the

sample concentration and dilute it to the measuring range with deionized water in the kit). A 5 mL kit of deionized water was used as a blank control.
(3) Potassium Persulfate reagent was added into reagent tube.
(4) Cover the lid and shake vigorously to dissolve the powder.
(5) Insert the test tube into the digestion device and heat digestion for 30 minutes.
(6) Immediately after digestion, take out the reagent tube and cool it to room temperature.
(7) Open the reagent tube and add 2 mL of 1.54N sodium hydroxide solution.
(8) Add 0.5 mL molybdovanadium heteropolysate reagent into the reagent tube and react for 7 minutes.
(9) After the time is over, clean the test tube and measure the reading.

2.3 DNA extraction methods

(1) Weigh 0.5g glass beads in the grinding tube, and add Buffer SLX MLUS 800 to the evenly mixed sample in advance, and shock it (speed 6.0, cycle 5, time 1min, interval 1min30s).
(2) Add 80 microliter Buffer DS, vortex uniform (10 s).
(3) 70°C water bath temperature for 10 minutes, including vortices every 5 minutes.
(4) Centrifuge 12000 g for 5 min. Transfer all the supernatant into the new 2 mL EP tube, and follow the kit instructions for remaining steps after sample pretreatment.
