# Supplementary material for: Nutrient availability contributes to structural and functional diversity of microbiome in Xinjiang oilfield
Source: Front Microbiol. 2024 Jul 31;15:1450226. doi: 10.3389/fmicb.2024.1450226 (PMC11322141; doi:10.3389/fmicb.2024.1450226)
Supplement: Supplementary file 2 [file Table_1.PDF]

Supplementary Tables

Table 1. The total mapped abundance to EggNOG database.

Sample name	Mapped Abundance-RPKs
7 days-10 Sampling Port	58.02%
20 days-10 Sampling Port	55.60%
39 days-10 Sampling Port	60.31%
42 days-10 Sampling Port	61.73%
48 days-10 Sampling Port	52.32%
53 days-10 Sampling Port	47.30%
65 days-10 Sampling Port	47.98%
71 days-10 Sampling Port	51.55%
99 days-10 Sampling Port	52.90%

Table 2. The major community composition identified by HuMAnN2.

Genus	7 days	20 days	39 days	42 days	48 days	53 days	65 days	71 days	99 days
unclassified	36.84%	30.64%	17.57%	17.36%	36.69%	50.10%	51.74%	46.01%	34.91%
Methanocorpusculum	0.00%	10.42%	15.35%	7.67%	15.64%	13.17%	12.22%	15.42%	19.12%
Vibrio	0.30%	9.25%	40.21%	44.97%	3.91%	3.14%	0.62%	0.15%	0.00%
Methanosarcina	0.01%	16.99%	8.13%	3.59%	5.73%	5.84%	2.12%	1.55%	0.42%
Enterobacter	23.95%	2.91%	2.05%	1.66%	1.76%	1.79%	2.39%	1.71%	0.40%
Marinobacter	0.23%	0.00%	0.95%	2.82%	11.22%	4.21%	7.33%	2.85%	7.70%
Pseudomonas	7.38%	0.74%	1.13%	1.39%	2.74%	2.42%	2.74%	6.64%	11.60%
Hyphomonas	0.60%	0.16%	0.83%	1.42%	3.29%	2.79%	4.33%	3.54%	3.64%
Desulfovibrio	3.92%	6.49%	2.63%	3.25%	0.70%	0.84%	1.17%	1.10%	0.00%
Klebsiella	5.22%	8.71%	0.93%	0.70%	0.92%	0.00%	0.00%	0.00%	0.00%
others	21.55%	13.70%	10.22%	15.17%	17.39%	15.70%	15.34%	21.03%	22.21%
